# Emergency Department-based Hepatitis A Vaccination Program in Response to an Outbreak

**DOI:** 10.5811/westjem.2020.4.45847

**Published:** 2020-07-10

**Authors:** Caroline Kaigh, Andrea Blome, Kraftin E. Schreyer, Megan Healy

**Affiliations:** * Cooper University Hospital, Department of Emergency Medicine, Camden, New Jersey; † Temple University Hospital, Department of Emergency Medicine, Philadelphia, Pennsylvania

## Abstract

**Introduction:**

The Philadelphia Department of Public Health (PDPH) declared a public health emergency due to hepatitis A in August 2019.^1^ Our emergency department (ED) serves a population with many of the identified risk factors for hepatitis A transmission. This study examines the impact of an ED-based hepatitis A vaccination program, developed in partnership with the PDPH, on incidence of hepatitis A infection and hospital admission.

**Methods:**

We conducted a retrospective review of all ED visits in the 12-week period centered around the implementation of the ED-based hepatitis A vaccination program. All adult patients presenting to the ED were offered vaccination, with vaccines supplied free of charge by the PDPH. We compared the incidence of diagnosis and of hospital admission for treatment of hepatitis A before and after implementation of the program.

**Results:**

There were 10,033 total ED visits during the study period, with 5009 of them prior to the implementation of the vaccination program and 5024 after implementation. During the study period, 669 vaccines were administered. Before the vaccination program began, 73 patients were diagnosed with hepatitis A, of whom 67 were admitted. After implementation of the program, 38 patients were diagnosed with hepatitis A, of whom 31 were admitted.

**Conclusion:**

A partnership between an ED and the local public health department resulted in the vaccination of 669 patients in six weeks in the midst of an outbreak of a vaccine-preventable illness, with a corresponding drop in ED visits and hospital admission for acute hepatitis A.

## INTRODUCTION

In August 2019, the Philadelphia Department of Public Health (PDPH) declared hepatitis A a public health emergency. At that time, 154 cases had been identified, compared to fewer than 10 cases reported annually in prior years.^1^

Populations at risk of acquiring hepatitis A include people experiencing homelessness, people who use drugs, men who have sex with men, and individuals recently incarcerated.^1^ The homelessness crisis in many major United States cities has previously been identified as a contributing factor in the increasing numbers of outbreaks of infectious diseases such as hepatitis A.^2^ Prior data from a similar outbreak in San Diego in 2018 demonstrated that 64.7% of patients infected with hepatitis A were homeless and 39.8% reported drug use.^3^

The emergency department (ED) where the intervention took place is located within the section of Philadelphia where many hepatitis A cases were being reported, and also serves the identified high-risk populations. The population includes a high volume of patients with substance use disorder, with the highest frequency of naloxone administration compared to other larger EDs in the city.^4^

Coordination between the PDPH, ED leadership, physicians and nursing, and pharmacists was required to develop the program. It took approximately one month from initial contact between the ED and PDPH until program go-live. Vaccines were supplied by the health department and stored in refrigeration units in both the hospital pharmacy and ED. All adult patients were asked by nursing during triage if they would like to be vaccinated for hepatitis A during their visit, given the local outbreak. Risks and benefits of the vaccine were reviewed at that time.

If patients consented to the vaccination, a best practice advisory (BPA) that contained the order for the health department-supplied vaccine was created in the patient’s chart and would automatically fire once the chart was accessed by a provider. The provider then ordered the vaccine to be administered free of charge. A recent study from the 2018 San Diego outbreak showed that utilization of a BPA within the electronic health record (EHR) helped identify high-risk patients and remind providers of vaccine availability, leading to an overall 77% vaccination rate of their target population.^5^

The Centers for Disease Control and Prevention recommends administering two doses of the vaccine, six months apart. However, a single dosage is up to 98% effective at preventing transmission, which is optimal for patients without primary care physician follow-up who present to the ED.^6^ The emergency physicians and public health department decided together that due to the urgency of the outbreak, the vaccination program would focus solely on getting the first of the series to as many patients as possible. To avoid the administration of repeated doses to a single patient, the BPA would not fire for patients who had already received the vaccine within the health system. We sought to evaluate the ED-based vaccination program and its impact on public health. Specifically, we tracked vaccine offered and administered, as well as suspected cases of hepatitis A presenting to the ED and patients admitted to the hospital for hepatitis A both before and after the intervention.

## METHODS

The vaccination program was implemented on August 27, 2019. A retrospective review of all visits to the ED spanning the vaccination program, from July 16–October 8, 2019, was conducted. ED visits were evaluated for ultimate diagnosis of hepatitis A and were compared before and after implementation of the vaccination program. The EHR was queried for the number of BPAs that fired once the vaccination program was implemented, as well as the number of vaccines administered.

## RESULTS

There were 10,033 total visits to the ED during the study period. Of these, 5009 visits preceded the vaccination program, while 5024 followed. The BPA fired on a total of 1164 ED patients. A total of 669 hepatitis A vaccines were administered between August 27, 2019–October 8, 2019. Before the vaccination program began, 73 patients were diagnosed with hepatitis A in the ED, 67 of whom were admitted. After the vaccination program was initiated, 38 patients were found to have a diagnosis of hepatitis A, of whom 31 were admitted. Results are summarized in Table 1.

## DISCUSSION

A recent hepatitis A outbreak in Philadelphia prompted a collaboration between the PDPH and our ED. The ED-based vaccination program was successful in vaccinating a large number of individuals in a short period of time. We observed a corresponding drop in both identified cases of hepatitis A and admissions for hepatitis A in the post-intervention period. The intervention demonstrated the importance of this collaboration in using the ED to improve population health.

We were fortunate to be able to partner with the PDPH, which supplied vaccines at no cost. The usual cost to our pharmacy of a hepatitis A vaccine is $58.40 per dose. While we did not collect data on costs associated with ED visits or admissions for treatment of hepatitis A either before or after our intervention, we believe that the savings associated with a reduction in incidence of hepatitis A far outweighs the total cost of vaccine administration.

It is likely that some of the drop in hepatitis A cases was the result of outside efforts from PDPH and other local organizations to get patients vaccinated, education of the public, and sanitation efforts. Several stand-alone vaccination events were held at nearby locations such as a busy needle exchange program. We were, however, able to vaccinate a large number of patients over a short period in an underserved area. Previous ED-based, hepatitis A vaccination programs have been reported, but have vaccinated smaller numbers of patients. For instance, a 2007 program in Boston vaccinated 122 patients. They targeted their intervention on a narrower group of patients experiencing homelessness, using drugs, or recently incarcerated.^7^

The ED will continue to provide vaccine beyond the reported time frame, until the outbreak is sufficiently addressed or vaccine is no longer available. The model has been adapted and scaled up to provide vaccination at a second, larger site within the same health system that serves a similar vulnerable population. The model has also been shared with other local EDs that are interested in developing ED-based, hepatitis A vaccination programs. To date, three other urban EDs are using this model to create their own programs.

A relevant question with this type of public health initiative is when and how vaccination programs should be used in an emergency setting. In our case, the PDHD identified hepatitis A as a sufficient threat to declare a state of emergency in the city. Many of the populations at risk have limited access to primary care. It has been previously reported that up to 73% of people experiencing homelessness identify at least one unmet health need.^8^ To reach these vulnerable groups, an innovative approach is required. When a local public health crisis is identified that affects populations with poor access to care, the ED may be considered as a potential setting for intervention.

## LIMITATIONS

Limitations of the study include the relatively small sample size as a result of intervention at a smaller site. While we saw a promising correlation between our intervention and a reduction in incidence of hepatitis A, we did not examine other parameters, such as effect on hospital admissions, changes in length of stay, or cost-effectiveness of a vaccination program. Our ED serves a large number of at-risk populations, including people experiencing homelessness and people with substance use disorder; the intervention may be less effective at sites with smaller proportions of these populations. We did not examine whether patients diagnosed with hepatitis A had previously received the hepatitis A vaccine, which may have confounded the effect of the intervention. We also were not able to estimate how much of the effect that we saw was secondary to outside public health efforts aimed at our patient population.

## CONCLUSION

A collaboration between a local ED and the public health department resulted in the vaccination of 669 patients in six weeks, in the midst of the outbreak of a vaccine-preventable illness. Stakeholders in the project included the ED leadership, physicians, nurses, pharmacists, and public health officials. The ability of the health department to furnish the vaccine free of charge and the support of the various stakeholders allowed a large number of patients in a high-risk area to be vaccinated quickly. We observed a corresponding drop in ED visits and inpatient hospitalizations for hepatitis. This model of collaboration between the ED and public health officials can be adopted by other departments experiencing outbreaks of vaccine-preventable illnesses, particularly those that affect high-risk populations that frequently use EDs for their care.

## Figures and Tables

**Table 1:** Analysis of emergency department (ED) visits, hepatitis A vaccines administered, diagnoses of, and admissions for, hepatitis A during the study period.

	Total ED Visits	Hepatitis A Vaccines Administered	Cases of Hepatitis A	Admissions for Hepatitis A n (% of cases)
Pre	5009	0	73	67 (91.8%)
Post	5024	669	38	31 (81.6%)

“Pre” includes data collected from July 16–August 26, 2019. “Post” includes data collected from the go-live date of August 27–October 8, 2019.
